# Adjuvant and neoadjuvant small-molecule targeted therapy in high-risk renal cell carcinoma

**Published:** 2009-05

**Authors:** A. Kapoor, A. Gharajeh, A. Sheikh, J. Pinthus

**Keywords:** Renal cell carcinoma, adjuvant therapy, neoadjuvant therapy, neo-adjuvant, review

## Abstract

**Background:**

Non-localized renal cell carcinoma (rcc) carries a poor prognosis with a significant risk of mortality for patients. Traditionally, interleukin-2 and interferon alfa have been administered in this setting, with high toxicity and limited improvement in cancer-specific survival. However, newer agents such as sunitinib, sorafenib, bevacizumab, and temsirolimus have demonstrated great potential and provide a new frontier in the management of high-risk rcc.

**Methods:**

We queried PubMed and the Medline ovid databases for English articles from 1950 to December 2008 using the keywords “renal cell carcinoma,” “high risk” and “renal cell carcinoma,” and “neoadjuvant.” Articles from these searches and the reference lists of relevant articles were obtained. Articles published between 1996 and 2008 were included in the present review.

**Results:**

Risk stratification is imperative for optimal patient selection in adjuvant, neoadjuvant, and research settings. Utilization of interferon alfa and interleukin-2 has not demonstrated improved disease-free survival in the adjuvant setting. A number of adjuvant vaccines have also failed to demonstrate improved survival. The adjuvant role of targeted small-molecule inhibitors such as sorafenib, sunitinib, and temsirolimus is currently under investigation in phase iii trials. Sporadic case reports have demonstrated promising results with neoadjuvant use of these agents, and a pilot study of neoadjuvant temsirolimus is currently underway at our centre.

**Conclusions:**

The role, efficacy, and toxicity of adjuvant and neoadjuvant targeted small-molecule inhibitors in high-risk rcc remains to be delineated. Ideally, clinicians will be able to identify high-risk patients and offer treatment to those who would benefit most from adjuvant and neoadjuvant therapy, while minimizing toxicity in low-risk patients.

## 1. INTRODUCTION

With more than 54,000 new cases and an estimated 13,000 deaths in the United States in 2008, renal cell carcinoma (rcc) shows a mortality rate that has declined slightly since 1990, but that continues to inflict a large burden of disease 1. Nephrectomy provides curative treatment for localized disease, but unfortunately, 30% of patients subsequently experience recurrence and metastasis, with survival rates below 10% 2. With accurate preoperative risk stratification, patients at high risk can be identified and offered neoadjuvant or adjuvant therapy for optimal management. The advent of small-molecule targeting agents such as sunitinib, sorafenib, and temsirolimus provides an avenue for such therapy in this patient population. We address such treatments here, and also discuss a pilot study currently under way at our centre to investigate neoadjuvant temsirolimus for high-risk rcc.

## 2. APPROACH TO NEOADJUVANT OR ADJUVANT THERAPY

### 2.1 Defining Risk

Although a complete discussion of the current status of risk stratification and prognostication in rcc is beyond the scope of this review, an accurate definition of risk in patients with rcc is imperative in determining those most likely to benefit from adjuvant and neoadjuvant therapy and in reducing toxicity in low-risk patients. We recommend a recent review by Downs *et al.* 3 for a more comprehensive discussion of this important and rapidly evolving topic of risk assessment in rcc.

Currently, tumour stage continues to be the most important prognostic factor for patients with rcc. This understanding has been validated in a study of 2746 patients followed for a median of 9 years: 5-year cancer-specific survival rates by stage were 97% (pT1a), 87% (pT1b), 71% (pT2), 53% (pT3a), 44% (pT3b), 37% (pT3c), and 20% (pT4), using the 2002 American Joint Committee on Cancer tumour classification system 4. Within the current pT3 classification, the level of inferior vena cava involvement or the type of tissue invaded has failed to be associated with a significant difference in survival, and Terrone *et al.* further classified pT3 tumours by defining those invading either the perirenal or sinus fat to significantly constitute the lowest mortality risk 5.

In addition to tumour stage, other parameters such as age, performance status, constitutional symptoms, number of metastatic sites, site of metastasis, sarcomatoid histology, papillary rcc type 2 histology, Fuhrman grade, microvascular tumour invasion, neutrophil count, serum lactate dehydrogenase level, serum C-reactive protein level, thyroid-stimulating hormone level, plasma adiponectin, oncofetal protein Imp3 (insulin-like growth factor ii mrna-binding protein 3), vascular endothelial growth factor (vegf), carbonic anhydrase ix, intratumoral polyamines, erythropoietin, B7-H1, and Ki-67 have illustrated prognostic and stratification utility in various studies 2,6–17. Further studies are required with larger patient numbers and longer duration of follow-up to delineate the role of these variables in the natural history of rcc, to identify limitations, and to establish external validation of findings.

Recently devised integrated stratification systems attribute a value to various clinical and histologic features, and these combinations permit risk assessment within a defined patient population. The two most extensively studied integrated stratification systems for rcc are the Mayo Clinic stage, size, grade and necrosis (ssign) score for clear-cell rcc (ccrcc) and the University of California–Los Angeles (ucla) integrated staging system (uiss) for rcc 18,19. The ssign scoring algorithm was devised following an analysis of 1801 patients with unilateral ccrcc. The analysis revealed that the 1997 TNM staging system, tumour size greater than 5 cm, nuclear grade, and histologic necrosis are predictive of cancer-specific mortality 18. Patients with ssign scores of 0–2, 3–4, 5–6, and 7–9 have 5-year cancer-specific survival rates of 100%, 91%, 64%, and 47% respectively; all patients scoring 10 or more die of their disease within 2 years 3.

The uiss system uses a combination of 1997 TNM stage, Fuhrman grade, and Eastern Cooperative Oncology Group performance status (ecog-ps) that was identified by Zisman *et al.,* through an analysis of 661 patients at ucla, as significantly predictive of cancer-specific survival 19. Initially, this method identified 5 statistically significant categories that stratified metastatic and nonmetastatic patients together, with 5-year survival in uiss categories i, ii, iii, iv, and v being 94%, 67%, 39%, 23%, and 0% respectively 19. These 5 categories were later incorporated into either metastatic or nonmetastatic low-risk, intermediate-risk, and high-risk stratifications, providing a practical means of assessing risk in patients with rcc not unlike the system used in prostate cancer 20. Reported 5-year disease-specific survival for low-, intermediate-, and high-risk nonmetastatic patients are 91%, 80%, and 54%; for metastatic patients, the corresponding rates are 32%, 20%, and 0%. To assign risk categories, decision boxes have been created for metastatic and nonmetastatic patients, in which risk is defined by progression downward from stage to grade to ecog-ps. In addition, analysis of freedom from recurrence in nonmetastatic patients revealed that 91% of low-risk, 64% of intermediate-risk, and 37% of high-risk patients are free from any recurrence at 5 years. Given these statistically significant differences in recurrence, and a tendency for low-risk patients to recur in the chest (high-risk patients recur in the abdomen), various postoperative surveillance regimens have been defined 21,22.

Both of these integrative models have been externally validated: the uiss with at least 8249 patients, and the ssign with 2656 patients 23–26. Regarding predictive capacity, the ssign score appears to be slightly superior to the uiss in nonmetastatic patients, having shown a predictive accuracy of 0.830 as compared with 0.760 in one study of 388 patients. However, the authors of the latter study did not report whether the difference was statistically significant, and clinical significance has yet to be established 27. Nevertheless, recent findings indicate that the addition of 5 molecular markers to the uiss increases its predictive accuracy to 0.903 27. The uiss system is currently limited by the complexity and technical demands of obtaining molecular markers from all patients. A potential limitation of the ssign system is its reliance on histologic tumour necrosis, which does not have a standardized definition, consensus for reporting, or availability at many centres 27. Lastly, it should be noted that the ssign is applicable only to patients with ccrcc (Table i).

Nomograms have also been developed to elucidate the prognosis of patients with rcc; however, although these tools are useful for individuals, they do not stratify patients into risk groups, thus limiting their role in clinical trial design and implementation of adjuvant and neoadjuvant therapy 28–31.

### 2.2 Adjuvant Therapy

Much effort has been invested toward the development of an effective and pragmatic strategy for adjuvant treatment of rcc. The use of radiation therapy was investigated in this light and found to be equivalent to observation in terms of relapse rate and survival 32,33. Furthermore, this modality expressed significant morbidity and mortality with a 44% complication rate 32. Currently, radiation therapy in the adjuvant setting has been abandoned and is being used only for palliation of symptomatic bone metastases.

The use of hormonal therapy has also been explored as a potential for adjuvant treatment of high-risk rcc. In a prospective randomized study of 136 patients, medroxyprogesterone acetate was found to provide no benefit with regard to disease recurrence and was associated with significant toxicity 34.

Immunotherapy has been another area of active investigation as an adjuvant strategy in rcc. A modest benefit in survival was reported with interferon alfa (ifnα) and with interleukin-2 (il-2) therapy in the context of metastatic rcc, but these immune modulators do not currently have a defined role in the adjuvant setting. In randomized trials, adjuvant ifnα and the widely available recombinant ifnα2b have been shown not to contribute to survival or relapse-free survival 35,36. For example, a phase iii trial investigating adjuvant il-2 in high-dose bolus form was closed early because an interim analysis revealed that disease-free survival was not affected 37. A subsequent study investigating adjuvant il-2 in low-dose subcutaneous form was also ineffective with respect to disease-free survival 38.

A more passive method of immunomodulation and its application to adjuvant therapy in rcc has come in the form of tumour vaccines. Galligioni *et al.* investigated the use of autologous irradiated tumour cells mixed with bacillus Calmette–Guèrin as an adjuvant strategy and found no statistically significant improvement in overall or disease-free survival 39, but a multicentre phase iii randomized controlled trial of adjuvant autologous tumour-cell vaccine conducted in Germany show a statistically significant disease-free survival benefit 40. In the latter investigation, 379 patients with pT2–3b pN0–3 M0 disease were included in the analysis, and 5-year progression-free survival was 77.4% and 67.8% in the vaccine and the control groups respectively. That study has been widely criticized because 174 patients were lost to follow-up after randomization and also because differences in overall survival were not analyzed.

Another vaccine strategy has focused on the use of heat shock proteins. The heat shock protein peptide complex HSPPC-96 (vitespen) was developed from autologous tumours in rcc. Following encouraging results in a phase ii trial, a 728-patient multicentre open-label randomized phase iii trial compared adjuvant HSPPC-96 with observation following nephrectomy and found no difference in recurrence-free survival after a median follow-up of 1.9 years 41.

The use of monoclonal antibodies has also been considered in the adjuvant treatment of rcc. Recently, an immunoglobulin G1 antibody known as cG250 (WX-G250) was found to bind carbonic anhydrase ix, which is a rcc-specific tumour antigen. A phase iii trial investigating the use of cG250 as compared with placebo following nephrectomy [the ariser (Adjuvant Rencarex Immunotherapy Trial to Study Efficacy in Nonmetastasized RCC) study] is currently in progress, with results expected in 2013 42.

A new frontier in the adjuvant treatment of rcc has focused on the use of small-molecule kinase inhibitors and anti-vascular agents. Although no substantial studies have yet justified the use of these agents in the adjuvant setting, sorafenib 43, sunitinib 44, and temsirolimus 45 have been established as appropriate treatment options in metastatic rcc. These agents are thought to work through a mechanism influencing the involvement of the tumor-suppressor *VHL* gene in the pathogenesis of rcc. Spontaneous inactivation of *VHL* in sporadic cases of rcc results in the overexpression of vegfs, platelet-derived growth factors (pdgfs) and hypoxia-inducible factors (hifs) 44. Sorafenib and sunitinib reduce tumour-cell proliferation and angiogenesis by acting as small-molecule inhibitors of multiple kinases; vegf receptors 1, 2, and 3; pdgf beta receptors; and fms-like tyrosine kinase 3 (Flt3), among others 46. Temsirolimus is a mammalian target of rapamycin kinase inhibitor and antagonizes cell growth and proliferation by disrupting intracellular signalling pathways. Furthermore, temsirolimus blocks hifa, which drives the downstream regulation of a number of pro-angiogenic factors 45.

Given the favourable effect of these agents in the treatment of metastatic rcc, several trials are now underway to evaluate their use as adjuvant therapies in high-risk surgically resectable rcc (Table ii). The assure trial is a multicentre double-blind randomized study examining 1332 patients who underwent nephrectomy for pT1b, G3–4; pT2–pT4; or any T stage with node-positive disease. Patients will be stratified into intermediate high-risk or very high-risk groups and then randomized for 1 year of oral sorafenib, sunitinib, or placebo. Very high-risk patients are those with grades 3–4 pT3a, any pT3a with adrenal involvement, or pT3b–4 and any N+. Intermediate high-risk patients are those with grades 3–4 pT1b, any pT2, or grades 1–2 pT3a without adrenal involvement. In addition to disease-free and overall survival, the study will investigate biomarkers, genetic mutations, dna methylation, and genetic polymorphisms as predictors of survival and therapeutic benefit. The investigation commenced in May 2006, and the estimated date of completion is April 2016. (For details, search for “NCT00326898” at www.clinicaltrials.gov/ct2/search)

Another ongoing adjuvant trial called the sorce trial is a multicentre double-blind randomized study with an estimated enrolment of 1656 patients with resected primary rcc at high- or intermediate-risk of relapse (defined by investigators as a Leibovich score of 3–11). Patients are to be randomized to receive either sorafenib for 1 year, sorafenib for 3 years, or placebo. The primary outcome measure is disease-free survival; secondary outcome measures are metastasis-free survival, overall survival, cost effectiveness, and toxicity. The sorce trial opened recruitment in June 2007 and is estimated to be completed by August 2012. (For details, search for “NCT00492258” at www.clinicaltrials.gov/ct2/search)

A third trial known as s-trac addresses the use of adjuvant sunitinib. With a multicentre double-blind randomized design, this study will compare 1 year of oral sunitinib with placebo in 290 patients at high risk of recurrence (based on uiss criteria) following nephrectomy. The primary endpoint of the study is disease-free survival, and secondary endpoints to be assessed will be overall survival, safety, and patient-recorded outcomes. The projected timeline for this investigation is July 2007 to March 2011 47. (For details, search for “NCT00375674” at www.clinicaltrials.gov/ct2/search)

### 2.3 Neoadjuvant Therapy

A review of the current literature failed to reveal any prospective studies in the field of neoadjuvant therapy for high-risk rcc. Until recently, cytokine-based immunotherapy had been the mainstay of systemic therapy in rcc, but its significant toxicity and poor primary tumour response are believed to have limited any investigations in the neoadjuvant setting 47. However, with the advent and subsequent success of small-molecule inhibitors for advanced rcc 39–41, the prospect of their application in a neoadjuvant strategy is provocative and exciting. Certainly, the theoretical benefits are intriguing and include tumour downstaging, reduction in pro-angiogenic factors, and possible response in the primary tumour. Several centres have published stimulating case reports demonstrating that neoadjuvant treatment with various permutations of sorafenib, sunitinib, and bevacizumab resulted in a reduction of primary tumour size 47–49, tumour thrombus 47,49, bulky lymphadenopathy 47,48, and metastatic lesions 47.

## 3. McMASTER UNIVERSITY EXPERIENCE

Currently, there is a paucity of knowledge on the biologic response of rcc to small-molecule inhibitors *in vivo.* This, along with the exciting potential of the application of these agents in the neoadjuvant setting of high-risk rcc patients, has compelled our centre to undertake a nonrandomized open-label outpatient pilot study investigating the use of temsirolimus before nephrectomy in patients with high-risk and metastatic rcc. Patients receive 25 mg temsirolimus by intravenous infusion on a weekly basis for 12 weeks before nephrectomy. Patients with metastatic disease continue to receive the same dose postoperatively for a maximum of 24 months, or until disease progression. A renal-mass biopsy and biomarker analysis is performed upon entry into the study. Efficacy will be evaluated using the Response Evaluation Criteria in Solid Tumors, and safety will be evaluated using the National Cancer Institute Common Toxicity Criteria for Adverse Events. The approximate duration of this study is 5 years, with an estimated enrolment of 20 subjects.

To date, 5 patients have been enrolled in the trial. Three patients have undergone uneventful laparoscopic radical nephrectomy. Patient 1 (10-cm renal mass with bulky adenopathy T2N2M0) is ned (no evidence of disease) at 6 months post-nephrectomy. Patient 2 (9-cm renal mass, bulky adenopathy, pulmonary metastases T2N2M1) is ned at 3 months post-nephrectomy. Patient 3 (7-cm renal mass, pulmonary metastases, T2N0M1) has recently undergone uneventful surgery, and follow-up computed tomography is pending. Patients 4 and 5 are receiving weekly temsirolimus, and surgery is planned.

These preliminary and early results suggest that neoadjuvant temsirolimus before radical nephrectomy for advanced rcc may induce disease regression post-surgery, and may even lead to disease resolution in patients with low-volume disease. Longer term follow-up is necessary to assess overall progression-free survival and overall survival.

## 4. CONCLUSIONS

In summary, the role, efficacy, and toxicity of adjuvant and neoadjuvant targeted small-molecule inhibitors in high-risk rcc remain to be delineated. Ideally, clinicians will be able to identify high-risk patients and to offer treatment to those who would benefit most from adjuvant and neoadjuvant therapy, while minimizing toxicity in low-risk patients.

## Figures and Tables

**TABLE I:** Comparison of the clinical stage, size, grade, and necrosis (ssign) score and the University of California–Los Angeles integrated staging system (uiss) integrated models of risk stratification

*Model*	*Parameters*	*Histology validation*	*External (*n*)*	*Patients*	*Limitations*
ssign	TNM stage, size, grade, necrosis	ccrcc	Yes	2656	Reliance upon subjective variable of necrosis
uiss	ecog-ps, Fuhrman grade, TNM stage	rcc	Yes	8249	Reduced predictive power in nonmetastatic patients

TMN = tumour size, metastasis, and nodal involvement staging system; ccrcc = clear-cell renal cell carcinoma; ecog-ps = Eastern Cooperative Oncology Group performance status; rcc = renal cell carcinoma.

**TABLE II:** Ongoing adjuvant therapy trials for high-risk renal cell carcinoma (rcc)

*Trial name*	*Principal investigator*	*Estimated accrual (*n*)*	*Treatment arms*	*Primary outcome*	*Estimated date of completion*
assure (Adjuvant Sorafenib or Sunitinib for Unfavorable Renal Carcinoma)	N.S. Blazer–Haas, *et al.* National Cancer Institute	1332	Sunitinib: orally every other day; 4 weeks on, 2 weeks rest, and 6 weeks placebo sorafenib; vs. sorafenib: orally twice daily; 6 weeks on, 4 weeks placebo sunitinib, and 2 weeks rest; vs. placebo	Disease-free survival	Apr 2016
sorce (Phase iii Randomized Double-blind Study Comparing Sorafenib with Placebo in Patients with Resected Primary rcc at High or Intermediate Risk of Relapse)	T. Eisen, Cancer Research UK at Cambridge Research Institute	1656	Sorafenib: orally twice daily; 1 year on and 2 years’ placebo; vs. sorafenib: orally twice daily; 3 years on; vs. placebo	Disease-free survival	Aug 2012
s-trac (Sunitinib Treatment of Renal Adjuvant Cancer)	Pfizer Inc.	290	Sunitinib: orally every other day; 4 weeks on, 2 weeks rest, and 6 weeks placebo sorafenib; vs. sorafenib: orally twice daily; 6 weeks on, 4 weeks placebo sunitinib, and 2 weeks rest) vs. placebo	Disease-free survival	Sep 2011

## References

[1] Jemal A, Siegel R, Ward E (2008). Cancer statistics, 2008. CA Cancer J Clin.

[2] Jiang Z, Chu PG, Woda BA (2008). Combination of quantitative IMP3 and tumor stage: a new system to predict metastasis for patients with localized renal cell carcinomas. Clin Cancer Res.

[3] Downs TM, Schultzel M, Shi H, Sanders C, Tahir Z, Sadler GR (2008). Renal cell carcinoma: risk assessment and prognostic factors for newly diagnosed patients. Crit Rev Oncol Hematol.

[4] Frank I, Blute ML, Leibovich BC, Cheville JC, Lohse CM, Zincke H (2005). Independent validation of the 2002 American Joint Committee on Cancer primary tumor classification for renal cell carcinoma using a large, single institution cohort. J Urol.

[5] Terrone C, Gontero P, Volpe A (2008). Proposal of an improved prognostic classification for pT3 renal cell carcinoma. J Urol.

[6] Phuoc NB, Ehara H, Gotoh T (2008). Prognostic value of the co-expression of carbonic anhydrase ix and vascular endothelial growth factor in patients with clear cell renal cell carcinoma. Oncol Rep.

[7] Cho KS, Choi YD, Kim SJ (2008). A comprehensive prognostic stratification for patients with metastatic renal clear cell carcinoma. Yonsei Med J.

[8] Dall’Oglio MF, Ribeiro–Filho LA, Antunes AA (2007). Microvascular tumor invasion, tumor size and Fuhrman grade: a pathological triad for prognostic evaluation of renal cell carcinoma. J Urol.

[9] Atzpodien J, Royston P, Wandert T, Reitz M, on behalf of dgcin—German Cooperative Renal Carcinoma Chemo-Immunotherapy Trials Group (2003). Metastatic renal carcinoma comprehensive prognostic system. Br J Cancer.

[10] Leibovich BC, Han KR, Bui MH (2003). Scoring algorithm to predict survival after nephrectomy and immunotherapy in patients with metastatic renal cell carcinoma: a stratification tool for prospective clinical trials. Cancer.

[11] Royston P, Reitz M, Atzpodien J (2006). An approach to estimating prognosis using fractional polynomials in metastatic renal carcinoma. Br J Cancer.

[12] Pinthus JH, Kleinmann N, Tisdale B (2008). Lower plasma adiponectin levels are associated with larger tumor size and metastasis in clear-cell carcinoma of the kidney. Eur Urol.

[13] Griffiths DF, Verghese A, Golash A (2002). Contribution of grade, vascular invasion and age to outcome in clinically localized renal cell carcinoma. BJU Int.

[14] Jiang Z (2007). Prognostic biomarkers in renal cell carcinoma. Expert Rev Mol Diagn.

[15] Rioux–Leclercq N, Turlin B, Bansard J (2000). Value of immunohistochemical Ki-67 and p53 determinations as predictive factors of outcome in renal cell carcinoma. Urology.

[16] Rioux–Leclercq N, Delcros JG, Bansard JY (2004). Immunohistochemical analysis of tumor polyamines discriminates high-risk patients undergoing nephrectomy for renal cell carcinoma. Hum Pathol.

[17] Michael A, Politi E, Havranek E (2007). Prognostic significance of erythropoietin expression in human renal cell carcinoma. BJU Int.

[18] Frank I, Blute ML, Cheville JC, Lohse CM, Weaver AL, Zincke H (2002). An outcome prediction model for patients with clear cell renal cell carcinoma treated with radical nephrectomy based on tumor stage, size, grade and necrosis: the ssign score. J Urol.

[19] Zisman A, Pantuck AJ, Dorey F (2001). Improved prognostication of renal cell carcinoma using an integrated staging system. J Clin Oncol.

[20] Zisman A, Pantuck AJ, Wieder J (2002). Risk group assessment and clinical outcome algorithm to predict the natural history of patients with surgically resected renal cell carcinoma. J Clin Oncol.

[21] Antonelli A, Cozzoli A, Zani D (2007). The follow-up management of non-metastatic renal cell carcinoma: definition of a surveillance protocol. BJU Int.

[22] Lam JS, Shvarts O, Leppert JT, Pantuck AJ, Figlin RA, Belldegrun AS (2005). Postoperative surveillance protocol for patients with localized and locally advanced renal cell carcinoma based on a validated prognostic nomogram and risk group stratification system. J Urol.

[23] Patard JJ, Kim HL, Lam JS (2004). Use of the University of California Los Angeles integrated staging system to predict survival in renal cell carcinoma: an international multicenter study. J Clin Oncol.

[24] Zigeuner R, Hutterer G, Chromecki T (2008). External validation of the Mayo Clinic stage, size, grade, and necrosis (ssign) score for clear-cell renal cell carcinoma in a single European centre applying routine pathology. Eur Urol.

[25] Han KR Bleumer I Pantuck AJ Validation of an integrated staging system toward improved prognostication of patients with localized renal cell carcinoma in an international population J Urol 2003 170 pt 12221 4 14634383 10.1097/01.ju.0000096049.64863.a1

[26] Cindolo L, Chiodini P, Gallo C (2008). Validation by calibration of the ucla integrated staging system prognostic model for nonmetastatic renal cell carcinoma after nephrectomy. Cancer.

[27] Ficarra V, Novara G, Galfano A (2008). The “stage, size, grade and necrosis” score is more accurate than the University of California Los Angeles integrated staging system for predicting cancer-specific survival in patients with clear cell renal cell carcinoma. BJU Int.

[28] Sorbellini M, Kattan MW, Snyder ME (2005). A postoperative prognostic nomogram predicting recurrence for patients with conventional clear cell renal cell carcinoma. J Urol.

[29] Kattan MW, Reuter V, Motzer RJ, Katz J, Russo P (2001). A postoperative prognostic nomogram for renal cell carcinoma. J Urol.

[30] Hupertan V, Roupret M, Poisson JF (2006). Low predictive accuracy of the Kattan postoperative nomogram for renal cell carcinoma recurrence in a population of French patients. Cancer.

[31] Cindolo L, Patard JJ, Chiodini P (2005). Comparison of predictive accuracy of four prognostic models for nonmetastatic renal cell carcinoma after nephrectomy: a multicenter European study. Cancer.

[32] Kjaer M, Iversen P, Hvidt V (1987). A randomized trial of postoperative radiotherapy versus observation in stage ii and iii renal adenocarcinoma. A study by the Copenhagen Renal Cancer Study Group. Scand J Urol Nephrol.

[33] Aref I, Bociek RG, Salhani D (1997). Is post-operative radiation for renal cell carcinoma justified?. Radiother Oncol.

[34] Pizzocaro G, Piva L, Di Fronzo G (1987). Adjuvant medroxyprogesterone acetate to radical nephrectomy in renal cancer: 5-year results of a prospective randomized study. J Urol.

[35] Messing EM, Manola J, Wilding G (2003). Phase iii study of interferon alfa-nl as adjuvant treatment for resectable renal cell carcinoma: an Eastern Cooperative Oncology Group/Intergroup trial. J Clin Oncol.

[36] Pizzocaro G, Piva L, Colavita M (2001). Interferon adjuvant to radical nephrectomy in Robson stages ii and iii renal cell carcinoma: a multicentric randomized study. J Clin Oncol.

[37] Clark JI, Atkins MB, Urba WJ (2003). Adjuvant high-dose bolus interleukin-2 for patients with high-risk renal cell carcinoma: a Cytokine Working Group randomized trial. J Clin Oncol.

[38] Majhail NS, Wood L, Elson P, Finke J, Olencki T, Bukowski RM (2006). Adjuvant subcutaneous interleukin-2 in patients with resected renal cell carcinoma: a pilot study. Clin Genitourin Cancer.

[39] Galligioni E, Quaia M, Merlo A (1996). Adjuvant immunotherapy treatment of renal carcinoma patients with autologous tumor cells and bacillus Calmette–Guèrin: five-year results of a prospective randomized study. Cancer.

[40] Jocham D, Richter A, Hoffmann L (2004). Adjuvant autologous renal tumour cell vaccine and risk of tumour progression in patients with renal-cell carcinoma after radical nephrectomy: phase iii, randomised controlled trial. Lancet.

[41] Wood C, Srivastava P, Bukowski R, on behalf of the C-100-12 RCC Study Group (2008). An adjuvant autologous therapeutic vaccine (HSPPC-96; vitespen) versus observation alone for patients at high risk of recurrence after nephrectomy for renal cell carcinoma: a multicentre, open-label, randomised phase iii trial. Lancet.

[42] Shuch B, Li Z, Belldegrun AS (2008). Carbonic anhydrase ix and renal cell carcinoma: prognosis, response to systemic therapy, and future vaccine strategies. BJU Int.

[43] Escudier B, Eisen T, Stadler WM, on behalf of the target Study Group (2007). Sorafenib in advanced clear-cell renal-cell carcinoma. N Engl J Med.

[44] Motzer RJ, Hutson TE, Tomczak P (2007). Sunitinib versus interferon alfa in metastatic renal-cell carcinoma. N Engl J Med.

[45] Hudes G, Carducci M, Tomczak P, on behalf of the Global arcc Trial (2007). Temsirolimus, interferon alfa, or both for advanced renal-cell carcinoma. N Engl J Med.

[46] Jacobsohn KM, Wood CG (2006). Adjuvant therapy for renal cell carcinoma. Semin Oncol.

[47] Shuch B, Riggs SB, LaRochelle JC (2008). Neoadjuvant targeted therapy and advanced kidney cancer: observations and implications for a new treatment paradigm. BJU Int.

[48] Amin C, Wallen E, Pruthi RS, Calvo BF, Godley PA, Rathmell WK (2008). Preoperative tyrosine kinase inhibition as an adjunct to debulking nephrectomy. Urology.

[49] Karakiewicz PI, Suardi N, Jeldres C (2008). Neoadjuvant Sutent induction therapy may effectively down-stage renal cell carcinoma atrial thrombi. Eur Urol.

